# Feasibility of a Broad Test Battery to Assess Physical Functioning Limitations of People Experiencing Homelessness

**DOI:** 10.3390/ijerph18031035

**Published:** 2021-01-25

**Authors:** Julie Broderick, Sinead Kiernan, Niamh Murphy, Joanne Dowds, Cliona Ní Cheallaigh

**Affiliations:** 1 Discipline of Physiotherapy, School of Medicine, Trinity College Dublin, University of Dublin, D08 W9RT Dublin, Ireland; sikierna@tcd.ie; 2 Department of Physiotherapy, St. James’s Hospital, D08 X4RX Dublin, Ireland; nimurphy@stjames.ie (N.M.); jdowds@stjames.ie (J.D.); 3 Department of Clinical Medicine, School of Medicine, Trinity Translational Medicine Institute, Trinity College Dublin, D08 W9RT Dublin, Ireland; nicheacm@tcd.ie; 4 Department of Infectious Diseases, St. James’s Hospital, Trinity College Dublin, D08 X4RX Dublin, Ireland

**Keywords:** homeless, physical function, frailty, strength, mobility

## Abstract

Background: People who are homeless experience poor health. Reflective of overall health and factors such as acquired injuries, physical ability or functioning is often low among people who are homeless, but there is a lack of consistency of measures used to evaluate this construct. The aim of this study was to evaluate the feasibility of a broad test battery to evaluate limitations in physical functioning among people who are homeless. Methods: This cross-sectional, observational study occurred in a hospital in Dublin, Ireland. We evaluated lower extremity physical function (Short Physical Performance Battery), falls risk (timed up and go), functional capacity (six-minute walk test), stair-climbing ability (stair climb test), frailty (Clinical Frailty Scale), grip strength (handgrip dynamometer) and muscular mass (calf circumference measurement) in a population of people experiencing homelessness admitted for acute medical care. The test completion rate was evaluated for feasibility. Results: The completion rate varied: 65% (Short Physical Performance Battery), 55.4% (timed up and go), 38% (six-minute walk test), 31% (stair climb test), 97% (Clinical Frailty Scale), 75% (handgrip dynamometer), 74% (calf circumference measurement)). Collectively, the most common reasons for test non-participation were pain (24.1%, *n* = 40), not feeling well or able enough (20.1%, *n* = 33), and declined (11%, *n* = 18). Conclusion: The feasibility of the test battery was mixed as test participation rates varied from 31% to 97%. Physical functioning tests need to be carefully chosen for people who are homeless as many standard tests are unsuitable due to pain and poor physical ability.

## 1. Introduction

Homelessness is a significant societal and global problem. A person experiencing homelessness is someone without stable housing who may live on the streets, in a shelter, in temporary accommodation, or in some other unstable or non-permanent situation [1]. It is estimated that there are 307,000 people experiencing homelessness in the U.K. [2], 550,000 in the U.S. [3], and 235,000 in Canada [4] at any one point. Homelessness has increased rapidly in Ireland since 2015 [5].

Homelessness profoundly affects health [2], and the mortality rate for homeless adults is almost four times higher than in the general population [6]. Recent data from the U.K. report a mean age of death among people who were homeless as 45 years for men and 43 years for women, in comparison with 76 and 81 years, respectively, in the general population [7]. People experiencing homelessness suffer a high burden of ill health and frequently have multiple chronic medical conditions [8,9] as well as mental illness and addiction issues [8]. Common chronic diseases such as chronic obstructive pulmonary disease, asthma, epilepsy, heart disease, and stroke are considerably more prevalent among people experiencing homelessness compared to housed individuals [10]. It has been reported that people who are homeless are 60 times more likely to present for unscheduled health care in the emergency department [11] and there is a higher rate of acute medical admissions [12]. Doran et al. showed that 70.3% of all hospitalisations of homeless people result in either an emergency department visit or readmission within 1 month of discharge, with the majority of hospital readmissions occurring within the first 2 weeks after hospital discharge [13].

People who are homeless and admitted for acute inpatient care may represent an especially vulnerable group with distinct care needs due to the complexity of their medical and social problems [14]. It is therefore recommended that clinicians screen this population for physical deficits so appropriate rehabilitation services can be initiated [15]. Due to the earlier onset of geriatric conditions [16] such as falls, poor balance, frailty, and poor mobility, outcomes commonly used to assess people who are homeless are often extrapolated from the geriatric setting [15], but with a much lower mean age [15], the usefulness of these measures is not fully known.

Identification and application of a suitable test battery may indicate a person’s ability to perform everyday tasks, their ability to live independently [8,16] and may help identify at risk individuals who need input that is more intensive. This would also target resource use and appropriate discharge planning [16] as well as providing insight into early signs of disability, poor health, and increased death risk [16,17].

The objectives of this study were to assess the feasibility of a comprehensive test battery to assess physical functioning limitations in adults experiencing homelessness.

## 2. Materials and Methods

This single-centre observational cross-sectional study occurred in St. James’s Hospital, which is a large university teaching hospital serving adults resident in the south inner city in Dublin, Ireland. It is estimated that approximately 1000 people are sleeping rough or in emergency accommodation within the catchment area of St. James’s Hospital [12]. The institutional review board of Tallaght University Hospital/St. James’s Hospital approved this study. All participants provided full written informed consent.

### 2.1. Test Battery

A team of expert physiotherapy and medical clinicians/academics devised the test battery by consensus. Considerations were to include tests that: (i) evaluated constructs of impairments, physical functioning and performance, (ii) were not burdensome in terms of time, (iii) required minimal resources in terms of cost and equipment, (iv) could be easy applied to the clinical setting, (v) would be applicable across a spectrum of functional levels, and (vi) displayed sound psychometric properties. The test battery chosen is summarised in Table 1.

### 2.2. Study Procedure

The clinical lead of the Inclusion Health Service, a consultant general physician (CNC), performed an initial eligibility screen of all inpatients registered as homeless using live daily updates of the Power BI software system from November 2018 to May 2019 in St. James’s Hospital. The European Typology on Homelessness and Housing Exclusion (ETHOS) definition of homeless was employed to register patients [33], which included those who were sleeping rough (those sleeping outside without cover); those living in emergency accommodation such as a hostel, night shelter, or bed and breakfast (B&B) accommodation; those living with family and friends (where possible, this was ascertained); or in a squat.

The route to admission for participants was unplanned self-presentations to the Accident and Emergency/Emergency Department, which in cases of medical necessity, unscheduled medical admission to an inpatient ward setting for acute care followed. In the inpatient ward setting, potential participants were flagged to SK and then definitively screened against the following criteria: (i) hospital inpatient, (ii) homeless, and (iii) >18 years. Exclusion criteria were: (i) insufficient level of English to follow instructions required for study participation (unless translator present); (ii) cognitive impairment, delirium, agitated state, or other reasons to a degree that precluded assessment; (iii) medical or orthopaedic reasons that would preclude ability to complete test battery; and (iv) confirmed pregnancy.

Suitable patients were given a participant information leaflet and verbal information about the study. Study information was read aloud and worded appropriately to accommodate participants with literacy issues. All participants provided written informed consent prior to participation in the study, and following a process of rolling consent, the participant could quit the assessment at any point. Participants voluntarily participated in this study and no remuneration was provided. Each test was explained briefly and demonstrated to the participant. If they were willing to proceed, each test was carried out in turn.

### 2.3. Statistical Analysis

Descriptive analysis was performed with the percentage compliance with each element of the test battery reported. The reasons for non-completion were recorded. The test feasibility index or rate was assessed as a percentage. This was calculated from the number of participants who were able to participate in the test battery divided by the total number of participants who completed the test. The feasibility rates were interpreted based on pre-specified feasibility rates identified by Wouters et al. [34]: <50%, not feasible; 50–75%, quite feasible; and >75%, feasible.

## 3. Results

**Figure 1:**
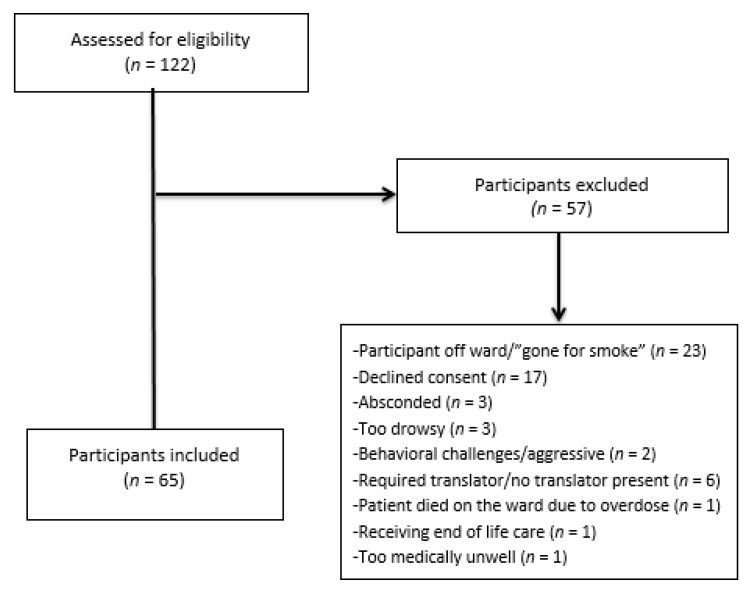
Flow diagram of participants through the study.

The flow of participants through the study is shown in Figure 1. Out of 122 patients assessed for eligibility, 57 were excluded for various reasons. The most prevalent reasons were that the patient was off the ward (*n* = 23) at the time or patient refusal (*n* = 17). In some cases, potential participants who were off the ward were recruited at a later time and thus included in the study numbers (*n* = 65). The results of the test battery are reported elsewhere (paper under review).

Participant demographics are presented in Table 2. The majority of participants (*n* = 44, 67.7%) were men and the median (IQR) age was 45 (38, 56) years with a range of 23 to 80 years. The majority of participants (*n* = 57, 87.7%) were born in Ireland. Most participants (*n* = 41, 64%) used hostel accommodation or were rough sleepers (*n* = 11, 17%). Eleven participants (16.9%) were re-admitted during the data collection period. More than half of the participants (*n* = 34, 52%) admitted to consuming excess alcohol. A smaller percent (*n* = 23, 35%) admitted to actively using heroin/intravenous drugs. Many participants suffered from pre-existing health conditions, with hepatitis (*n* = 27), liver disease (alcohol related) (*n* = 13), epilepsy/seizure disorders (*n* = 11), and mental health conditions (*n* = 17) being among the most common.

### 3.1. Compliance with Physical Performance Battery

Participants completed some or all of the outcome measures outlined in the test battery, as shown in Table 3. The entire test battery took approximately 30 min to complete and was completed in the same day where possible or within the same inpatient stay.

### 3.2. Completion Rates of Performance-Based Measures

The quite feasible [34] performance-based tests were the Short Physical Performance Battery (SPPB) (completion rate 65%, *n* = 42) and Timed Up and Go (TUG) (completion rate 55%, *n* = 36). The Six-Minute Walk Test (6MWT) and Stair Climb Test (SCT) were deemed not feasible” tests [34] with completion rates of 38% (*n* = 25) and 31% (*n* = 20), respectively.

### 3.3. Completion Rates of Tests Completed by Assessor

The completion rate of tests performed by the assessor was higher than performance-based tests. The Clinical Frailty Scale (CFS) was a highly feasible test [34], which was completed by the majority of participants (97%, *n* = 63). Measurement of calf circumference was quite feasible [34] with a completion rate of 74% (*n* = 48).

### 3.4. Reasons for Non-Completion

The specific reasons for non-completion of each test are outlined in Table 3. Collectively, the main reasons for non-completion were pain (24.4%, *n* = 40), not feeling well enough (20.1%, *n* = 33), and declined (11%, *n* = 18).

## 4. Discussion

This appears to be the first study to evaluate a broad physically focused test battery in hospital in-patients who were registered as homeless. In our sample, the median age was 45 years and 67.7% were men. This is broadly comparable to other cohorts of inpatients who were homeless with an average 46 years of age and 76.1% men [35] from a U.S. study, and 48 years and 76.5% men [36] from a Spanish study. A striking finding was the inability of many participants to conduct simple standard physical tests due to pain and poor physical ability.

No physical-focused outcomes [15] have been validated and no core outcome set exists [37] specific to people who are homeless. In this study, we piloted a comprehensive experimental test battery using standard clinical and psychometrically sound tests, mainly extrapolated from the geriatric setting. We chose tests that could be easily applied in the ward-based setting, require no specialist equipment, and be easily interpreted. Despite this careful planning at the outset of this study, not all evaluation tools were feasible for use.

We found that pain was the most common reason (24.4%) that precluded participation, highlighting the possible under-treatment of pain in this cohort. We also found that many standard geriatric tests were too challenging for this group to perform, despite a low median age of 45 years. Performance-based tests such as the SCT (31%) and 6MWT (38%) were not feasible [34]. Tests that were completed by the study assessor, such as the CFS (data generated for 97% of participants) and measurement of calf circumference (data generated for 74% of participants) were much more feasible [34]. There was also a higher level of feasibility [34] in low-threshold tests such as handgrip dynamometry (75%). Due to the process of rolling consent, participants could decline to participate in a test at any time, yet declining to complete the test only applied in 11% of cases.

Positive aspects of the test battery were the duration of testing (20–30 min), which did not appear to be overly burdensome to participants. Tests were easily conducted in the clinical environment and were safe as evidenced by the lack of adverse effects, but a qualified physiotherapist conducted all tests and assessed whether participants were suitable for test participation. This indicates the need to optimise pain and refine physical evaluation tools in this cohort.

Based on the results of this study, to optimise the test battery for further use, we propose a quick standardised test battery outlined in Table 4 below that could be applied to the clinical setting and future research studies. Tests might also be useful in a primary care setting where intervention programmes could be implemented that might improve physical status. To the best of our knowledge, this is the first time that a battery of tests has been proposed for the evaluation of impairments, physical functioning, and performance among people experiencing homelessness. In recommending these tests going forward, we concede there may be a ceiling effect for a small number with high functional capacity [38,39]; therefore, close evaluation of whether tests are applicable across the spectrum of functioning [15] would be required. The next step would be to assess the feasibility, validity, reliability, and sensitivity to detect change of this test battery and establish cut-off points to identify high-risk patients. In addition, as only 38% were able to walk for 6 min, the feasibility of other tests should be investigated such as the 1 min sit-to-stand test, which has been used in Chronic Obstructive Pulmonary Disease (COPD) populations [40], and gait speed over a 6 m course [41]. As pain was the main reason for not participating in tests, we recommend that pain is screened and optimised before the conduction of any physical tests in clinical or research settings. Although the proposed test battery would not be onerous in terms of time, we recommend that if testing could not be completed in its entirety in one session due to reasons such as fatigue or difficulties maintaining focus on physical tasks, that it could be conducted over a number of sessions within a meaningful time period (e.g., single hospital admission).

Contextual factors may also be important to consider in the interpretation of this study. Firstly, homeless populations in the U.S. include a high proportion of veterans and ethnic minorities, while the study cohort in Dublin, Ireland, reflecting previous work, includes predominantly white Irish participants and very few war veterans [12]. For instance, in our study >90% were white, which is higher than the U.S. study of hospitalised homeless, which included 62% white people [35]. A second major difference is that publicly-funded free primary and secondary healthcare is available to those falling into the lowest one-third income bracket in Ireland, so insurance status is not a factor limiting inpatient hospital care. Thirdly, in Dublin, homelessness is closely linked to drug use: up to 70% of homeless individuals report having used illegal drugs, many with poly drug use, and >50% report injecting drugs [42]. Approximately 70% of homeless individuals in Dublin consume alcohol at dangerous levels [42]. This reflects that diseases related to alcohol and drug use (abscesses, hepatic failure and haematemesis) as well as seizures are also more common among homeless inpatients in Ireland, which may result from the increased rate of traumatic brain injury and substance use in this population [12]. It is not known whether this profile limits the interpretation of results in other settings.

Other limitations to consider were that participants experienced a burden of physical and medical conditions that may have interacted with testing, which is an inherent limitation of evaluating a physical test battery in an acute hospital population. The exact reason for unscheduled medical admission was not recorded for the purposes of the present study. A previous detailed analysis of homeless inpatients (*n* = 459) within our centre [12] revealed that 94.9% of unscheduled medical admissions were due to physical health needs including pneumonia/bronchitis (11.8%), seizures (8.5%), syncope and collapse (5.7%), acute exacerbation of COPD/asthma (5.3%), abscess (5.0%), cellulitis (4.8%), venous thromboembolism (3.5%), haematemesis (3.27%), hepatic failure (2.18%), and alcohol withdrawal (2.18%). It is likely that a similar pattern pertained to the present study. Acute or chronic presentation of one or more of these conditions or acute trauma may have influenced mobility levels at the time of testing and therefore the ability to participate in the test battery, the extent to which is difficult to elucidate. A further limitation was that this study was subject to selection bias, as all participants were recruited as hospital inpatients. Data were collected from one urban hospital setting, but as deficits in physical functioning ability are prevalent [15] among a range of homeless settings, results may be cautiously applicable to homeless shelters and hostels, but this requires further evaluation. We concede that comparing other non-homeless hospitalised patients matched by factors such as age, sex, and co-morbidities would provide a useful objective comparison, but this was beyond the scope of the present study. This should be a focus for a follow-up study. Finally, we excluded participants with severe impairment who were unable to complete the majority of the test battery due to orthopaedic or medical reasons. Therefore, this study may unwittingly be a snapshot of participation levels in those with less-severe physical ability.

The strengths of this study were the application of psychometrically sound measures to evaluate constructs of impairments, physical functioning and performance. A qualified physiotherapist familiar with the testing battery performed all tests and followed standardised methodology. In addition, a reasonable sample size to gather perspectives on the feasibility of these measures was generated in this study.

## 5. Conclusions

To assess physical ability, it is necessary to use appropriate tools that are context-specific to the population under evaluation. We found the most feasible tests were the CFS, handgrip dynamometry, and calf circumference measurement. The 6MWT and SCT were not feasible tests for use in this cohort. Based on results of this study, we propose a test battery that may be feasible for use in this population. This requires further evaluation, but may be useful for research and clinical studies to more closely investigate physical functioning limitations in people who are homeless.

## Figures and Tables

**Table 1:** Summary of test battery.

Test	Construct Measured	Performance-Based Measure/Assessed by Tester	Test Description	Scoring/Unit of Measurement	Interpretation	Reference/Comparative Values
Short Physical Performance Battery (SPPB) [18]	Lower extremity physical function	Performance-based measure	Consists of 3 tasks: (i) a balance task, (ii) 5 timed chair stands, (iii) a short timed walk	0–12	Higher scores indicated better performance	<10: indicates one or more mobility limitations [19]
Timed Up and Go (TUG) [20]	Falls risk [21]	Performance-based measure	Measures the time it takes a person to stand up from an armchair, walk three metres and turn back to return to the chair	Timed test (s)	Higher scores indicate worse performance	>20 s indicates low mobility [22] >14 s indicates high falls risk [23]
Six-Minute Walk Test (6MWT)	Functional capacity	Performance-based measure	Measures the distance covered up and down a 30 m course, over a six-minute period [24]	Distance covered (m)	Higher scores indicate better performance	Mean (SD) 6MWT distance in healthy subjects aged 55–75 years reported as 659 (62) m, with a range 484–820 m [25]
Stair Climb Test (SCT) [26]	Stair climb ability	Performance-based measure	Time taken for participants to ascent and descent stairs with 11 steps [27]	Time (seconds per step)	Low score indicates better performance	A sample of 106 elderly people with symptomatic hip or knee osteoarthritis completed the 11-step SCT with a time of 1.14 s per step [26]
Clinical Frailty Scale (CFS) [28]	Frailty	Assessed by tester	Each point on the scale is correlated with a description of frailty along with a visual chart to aid the tester in classifying frailty	1 (very fit) to 9 (terminally ill)	Higher scores indicate higher levels of frailty	Prevalence of frailty 10.7% in adults aged ≥65 years and increases to >50% in those ≥80 years of age [29]
Digital Hand Dynamometer [30]	Grip strength	Performance-based measure	Performed in a sitting position while the hand was unsupported with the elbow at 90° flexion and the underarm and wrist in neutral positions. Three measurements performed with each hand. An average of highest value for right and left sides is used for analysis.	Dynamometer score (kg)	Higher scores indicate better strength	Reference average handgrip strength values [31] for men aged 30–49 are 54 kg and women are 34.5 kg, and for 65–69 years of age, average handgrip strength are 44 kg for men and 28 kg for women [31]
Muscular Mass	Calf circumference measurement	Assessed by tester	Girth of mid-point calf circumference measured	Width (cm)	Higher score indicates higher levels of muscular mass	The cut-off for decreased muscle mass in the elderly [32] has been identified as 34 cm for men and 33 cm for women

**Table 2:** Demographic characteristics of participants.

Variable	*N*	%
Sex		
Male	44	66.7
Female	21	32.3
Race/Ethnicity		
White Irish	57	87.7
White non-Irish	5	7.7
African	2	3.1
Asian	1	1.5
Medical conditions		
Pancreatic disorders	6	9.3
Orthopaedic disorders	6	9.3
Chronic obstructive lung disease	3	4.6
Liver disease (alcohol related)	13	20
Skin disease	5	7.7
Ulcers	5	7.7
HIV	7	10.8
Hepatitis	27	41.5
Seizures	11	16.9
Amputee	4	6.1
Depression	10	15.4
Bipolar disorder	4	6.1
Schizophrenia	3	4.6
Other mental disorder	2	3.0
Current living arrangement		
Hostel accommodation	41	64.1
Rough sleeping	11	16.9
With family/friends	5	7.7
Sheltered accommodation	3	4.6
Hotel	3	4.6
Rehabilitation facility	1	1.5
Unknown	1	1.5

HIV: human immunodeficiency virus.

**Table 3:** Numbers of participants who completed physical test battery.

Construct Measured	Test	Completed *N* (%)	Women Completed *N* (%)	Men Completed *N* (%)	Reasons for Non-Completion
Lower extremity function	Short Physical Performance Battery (SPPB)	42 (65)	9 (56)	33 (75)	Pain (*n* = 8), not feeling able/well enough (*n* = 5), declined (*n* = 5), non-weight bearing (*n* = 3), no appropriate footwear (*n* = 2)
Falls risk	Timed up and go (TUG)	36 (55.4)	9 (43)	27 (61)	Pain (*n* = 10), declined (*n* = 7), no appropriate footwear (*n* = 2), not feeling able/well enough (*n* = 5), non-weight bearing (*n* = 2), reasons not stated (*n* = 3)
Functional capacity	Six-minute walk test (6MWT)	25 (38)	9 (43)	18(41)	Not feeling able/well enough (*n* = 13), pain (*n* =10), suicidal ideation (*n* = 2), no appropriate footwear (*n* = 2), non-weight bearing (*n* = 3), reasons not stated (*n* = 2)
Stair-climbing ability	Stair climb test (SCT)	20 (31)	5 (31)	15 (34)	Unable/unsafe to do so (*n* = 41), feeling dizzy (*n* = 1), pain (*n* = 2), afraid of stairs (*n* = 1)
Frailty	Clinical Frailty Scale (CFS)	63 (97)	21 (100)	42 (95)	Lack of information to rate scale (*n* = 2)
Grip strength	Handgrip dynamometry	49 (75)	16 (76)	33 (75)	Not feeling able/well enough (*n* = 5), pain (*n* = 5), fatigue (*n* = 3), suicidal ideation (*n* = 1), broken wrist (*n* = 2)
Muscular mass	Calf circumference	48 (74)	15 (88)	34 (77)	Not feeling able/well enough (*n* = 5), pain (*n* = 5), declined (*n* = 6), suicidal ideation (*n* = 1)

**Table 4:** Proposed test battery to evaluate physical functioning limitations in people who are homeless.

Test	Construct Measured	Reason for Choice/Caution
CFS	Frailty	High feasibility, completed by tester
Handgrip dynamometry	Strength	Low-threshold test to maximise participation
Calf and upper limb circumference	Muscle mass	To allow for lower limb swelling, also measure upper limb
SPPB	Physical performance/falls risk	Likely ceiling effect for a small number of high-level performers

## Data Availability

Not applicable.
